# Efficacy of pamidronate in children with chronic non-bacterial osteitis using whole body MRI as a marker of disease activity

**DOI:** 10.1186/s12969-019-0340-7

**Published:** 2019-07-04

**Authors:** C. S. Bhat, M. Roderick, E. S. Sen, A. Finn, A. V. Ramanan

**Affiliations:** 1 Departments of Paediatric Rheumatology and Immunology, Bristol Royal Hospital for Children, Level 6, Education Centre, Upper Maudlin Street, Bristol, BS2 8BJ UK; 2 Bristol Children’s Hospital and Translational Health Sciences Bristol Medical School, University of Bristol, BS81QU, Bristol, UK; 3 Department of Paediatric Rheumatology, Great North Children’s Hospital, NE14LP, Newcastle upon Tyne, UK

**Keywords:** Whole body MRI, Pamidronate, Spinal lesions

## Abstract

**Background:**

To study the response to pamidronate using whole body magnetic resonance imaging (WB-MRI) in children with chronic non-bacterial osteitis (CNO) in a tertiary health centre.

**Methods:**

The medical records of children under the age of sixteen with a diagnosis of chronic non-bacterial osteitis between 2005 and 2018 were reviewed. All those who were treated with pamidronate were included and relevant data was collected. Response to therapy was determined based on the status of lesions on WB- MRI.

**Results:**

Forty six patients were included in the study. Pre- and post-treatment WB-MRI was available in forty patients. Cumulative lesions pre-treatment were 150 and reduced to 45 (30%) post-treatment. Seventeen patients (42.5%) had a good response with complete resolution of all lesions and nine patients (22.5%) worsened during or following treatment with pamidronate. Vertebral disease had a good response and 82.3% of the lesions resolved completely.

**Conclusion:**

Our study describes the experience with pamidronate in a tertiary health centre using WB-MRI as a marker of disease activity. Pamidronate was well tolerated in our cohort and treatment response was fairly good.

**Significance and innovation:**

1. Bisphosphonates can be used in the treatment of CNO when response to NSAIDs is suboptimal. 2. In the presence of spinal or mandibular lesions bisphosphonates were used as first line. 3. Treatment was escalated to a TNF blocker when response to bisphosphonates was suboptimal.

## Background

Chronic non-bacterial osteitis (CNO) is an autoinflammatory disorder that predominantly affects metaphyses of long bones. Bone pain and bone swelling are the usual presenting symptoms [1]. Whole-body magnetic resonance imaging (WB-MRI) is becoming increasingly important for diagnosis and assessing treatment response [2]. Non-steroidal anti-inflammatory drugs (NSAIDs) are usually preferred first-line agents across most centres. Second-line options include non-biologic disease-modifying anti-rheumatic drugs (DMARDs), bisphosphonates and anti-tumour necrosis factor (TNF) agents. Initiating appropriate treatment with timely escalation is necessary to prevent complications especially with vertebral or mandibular disease [3].

Apart from the Euro fever registry that included 486 patients [4], studies pub lished so far highlighting the effectiveness of bisphosphonates in CNO have included only small numbers of patients [5, 6]. We report our experience of children with CNO treated with bisphosphonates over a 13-year period.

## Methods

In this retrospective study, medical records of patients diagnosed with CNO between 2005 and 2018 were reviewed. Children with age of disease onset less than 16 years who received bisphosphonates were included in the study. Demographics, clinical features, investigations and treatment received were recorded. WB-MRI was used to identify sites of bone and soft tissue inflammation in active CNO.

MRIs were acquired from clinical 1.5 T scanners (Siemens, Erlangen, Germany). All WB-MRIs included in the study had coronal two-dimensional fast STIR T2 and T1 sequence images. Dedicated axial images were obtained from specific sites depending on abnormalities identified on the coronal images. If required, contrast-enhanced MRIs were obtained after 0.1 mmol/kg of intravenous gadolinium. According to the Bristol diagnostic criteria [7], typical short tau inversion recovery (STIR) MRI changes include bone marrow oedema and/or bone expansion, lytic areas and periosteal reaction. Scans were reported by one of the five paediatric radiologists with musculoskeletal expertise at our centre. Response to therapy based on the status of lesions on WB-MRI was defined as:

- Good response - complete resolution of all lesions.
- Moderate response - combination of completely resolved and significantly resolved or stable lesions.
- Mild response - partial improvement of existing lesions or stable lesions.
- No response - combination of new lesions or worsening of existing lesions and stable lesions.

In the absence of vertebral or mandibular disease all patients had a trial of NSAIDs for at least a month. Treatment was escalated if the desired clinical response was not achieved and bisphosphonates were the preferred second line agents. If the response to bisphosphonates was suboptimal treatment was switched to a TNF blocker. Bisphosphonates were used as first line along with NSAIDs in those with mandibular or vertebral disease.

Earlier, the practice was to use a short course of corticosteroids for 4 to 6 weeks or/and methotrexate for a period of 18 to 24 months prior to bisphosphonates but this is no longer followed. In the subset that received corticosteroids or/and methotrexate whole body MRI was repeated before escalating treatment.

In our centre pamidronate was the preferred bisphosphonate. The dose of pamidronate varied depending upon clinical response but was usually given as 3-monthly cycles for a period of 12 months. Each cycle consisted of 3 days of pamidronate at a dose of 1 mg/kg/day and a maximum of 60 mg/day. The maximum cumulative dose was 12 mg/kg/year. Additional doses were given for persistent disease at the end of 12 months. Clinical response to treatment was assessed after the first two cycles. Patients who reported no improvement or had worsening of symptoms underwent a WB-MRI to assess disease activity. Scans were otherwise obtained at diagnosis and prior to escalation of treatment. In the subset that received pamidronate WBMRI was usually repeated after completion of four cycles.

## Results

Forty-six patients were included in the study and mean follow up duration was 6 years (range 1–13 years). Baseline characteristics are summarised in Table 1.

**Table 1:** Baseline characteristics of patients

Female: Male	3.18: 1	
Median age at diagnosis (IQR, years)	11.6 (10–13)	
Median time to diagnosis (IQR, months)	12 (5–24)	
Clinical characteristics (%)	Bone pain	36 (78.3)
	Bone swelling	10 (21.7)
	Synovitis	3 (6.5)
	Arthritis	6 (13)
	Pustulosis	3 (6.5)
	Psoriasis	3 (6.5)
	IBD^a^	1 (2.2)
Family history of psoriasis (%)	7 (15.2)	
Family history of IBD (%)	2 (4.3)	
Bone biopsy (%)	29 (63)	
Lesions pre-treatment on WB-MRI	162	
Treatment (%)	NSAIDs	35 (76)
	Pamidronate	46 (100)
	Corticosteroids	4 (8.7)
	Methotrexate	5 (10.9)
	TNF blockers (*n* = 40)	5 (12.5)

^a^*IBD* Inflammatory bowel disease

**Fig. 1:**
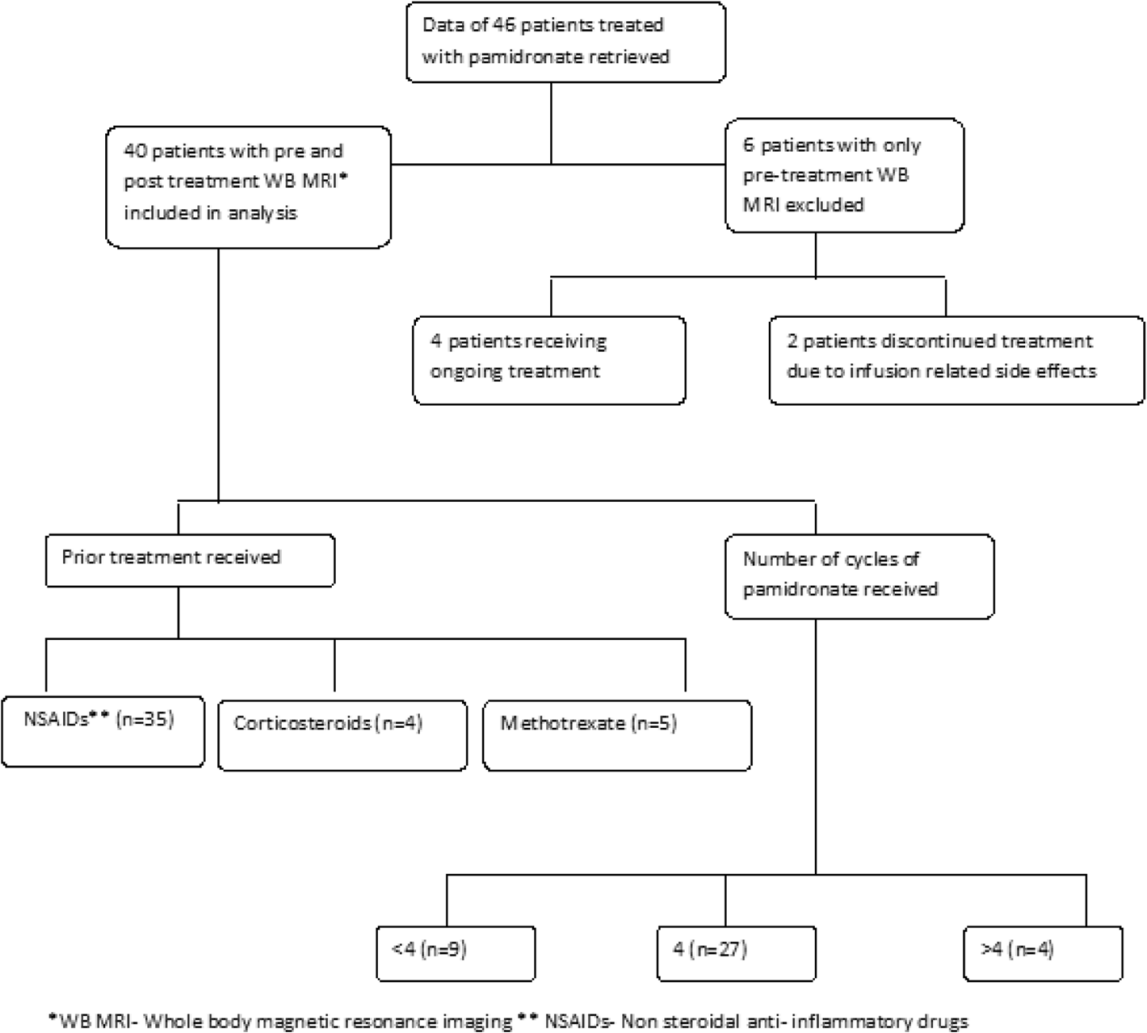
Flowchart summarising patient selection and treatment options used prior to pamidronate

Pre-treatment WB-MRI was performed in all patients and post-treatment WB-MRI was obtained in 40/46. Cumulative lesions pre-treatment were 162. Prior to being treated with pamidronate, thirty five patients had a trial of NSAIDs, four patients (8.7%) received corticosteroids and five (10.9%) had a trial of methotrexate. Treatment was escalated to pamidronate due to poor clinical or radiological response. NSAIDs along with bisphosphonates were used as first line in nine patients (19.5%) with spinal lesions and two patients (4.3%) with mandibular disease. Two patients discontinued treatment due to infusion related side effects and four patients are receiving ongoing treatment. Treatment response was assessed in those with a post-treatment WB-MRI (*n* = 40, lesions = 150). Data collection has been summarised in Fig. 1.

Twenty-seven patients (67.5%) completed 4 cycles of pamidronate. Four patients (10%) required extra doses of pamidronate because of persistent symptomatic disease. Nine patients (22.5%) discontinued treatment after two cycles due to poor clinical response. Treatment responses are summarised in Tables 2 and 3. Five patients (12.5%) with radiological evidence of persistent disease or worsening of disease were commenced on a TNF blocker.

**Table 2:** Treatment response to pamidronate

Response	Number of patients (%) (*n* = 40)	Lesions pre- pamidronate	Lesions post-pamidronate	Number resolved (%)
Good response	17 (42.5)	37	0	37 (100)
Moderate response	10 (25)	47	12	35 (74.5)
Mild response	4 (10)	18	6	12 (66.7)
No response	9 (22.5)	48	27	21 (43.7)

**Table 3:** Status of lesions pre- and post- pamidronate

Number of cycles	Number of patients (*n* = 40)	Lesions pre-treatment (*n* = 150)	Lesions post-treatment (*n* = 45)	Lesions resolved	Lesions persistent	Lesions worsened	New lesions
< 4	9	33	15	26	3	4	8
4	27	93	22	80	8	5	9
> 4	4	24	8	19	3	2	3

**Fig. 2:**
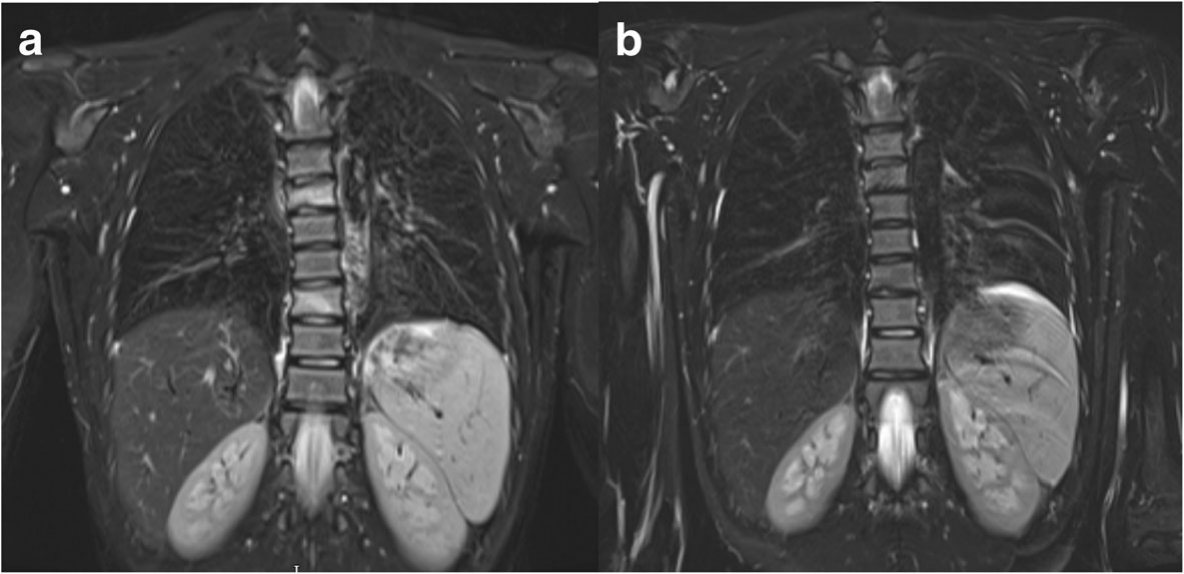
Pre and post-pamidronate WB-MRI. **a** The coronal STIR image shows high signal predominantly of the T6 and T9 vertebral bodies consistent with bone oedema. **b** Almost complete resolution of high signal changes in keeping with treatment response after four cycles of pamidronate. There is moderate loss of height at T6 and slight loss of height at T9 vertebral bodies

Nine patients had vertebral disease at presentation and 8/9 have completed treatment. Seventeen lesions were detected prior to treatment with pamidronate of which 14 (82.3%) resolved completely (Fig. 2) but 3 (17.6%) lesions persisted. One patient with collapsed vertebrae received additional doses of pamidronate. Despite this, follow-up imaging showed persistent disease and the child was eventually commenced on adalimumab. Another patient with reduced vertebral height had stable disease after four cycles of pamidronate. One patient developed sacroiliitis on follow up and was commenced on adalimumab. Distribution of lesions has been summarised in Table 4. Two patients had mandibular disease at presentation and were commenced on pamidronate. One patient discontinued treatment and post-pamidronate WB-MRI is pending in the other patient.

**Table 4:** Distribution of lesions

Location	Pre pamidronate	Post pamidronate	Number resolved (%)
Distal tibial metaphyses	22	7	15 (68.2)
Distal femur metaphyses	17	2	15 (88.2)
Clavicle	16	1	15 (93.7)
Proximal tibial metaphyses	15	5	10 (66.7)
Pelvis	11	3	8 (72.7)
Vertebra	17	3	14 (82.3)
Proximal femur metaphyses	8	0	8 (100)
Humerus	5	2	3 (60)
Talus	6	0	6 (100)
Calcaneus	5	0	5 (100)
Ulna	5	0	5 (100)
Scapula	5	0	5 (100)
Radius	4	0	4 (100)
Ribs	4	2	2 (50)
Navicular	4	0	4(100)
Mandible	2	0	2(100)
Fibula	1	0	1(100)
Metacarpals	1	0	1(100)
Metatarsals	1	0	1(100)
Sternum	1	0	1(100)

## Discussion

In our cohort, the number of radiologically apparent lesions began at 150 and fell to 45 (30%) after treatment with pamidronate. 82.3% of vertebral lesions resolved completely. This is in keeping with other published studies demonstrating efficacy of pamidronate [5, 6, 8–10].

Bisphosphonates have preferential affinity for sites of active bone remodelling such as bone inflammation as is seen in CNO. They limit both osteoblast and osteocyte apoptosis, reduce bone resorption and increase secondary mineralisation of bone. Suppression of bone resorption is maximum within 3 months of initiation of therapy and is more rapid with intravenous than oral therapy [11]. Pamidronate is hence the preferred bisphosphonate in our centre. The effects of pamidronate can be hampered in those with vitamin D deficiency [11, 12]. In our cohort, Vitamin D levels were routinely checked and corrected prior to treatment.

In the group that discontinued treatment due to poor clinical response, 50% of the lesions had improved on follow up WB-MRI. This highlights the importance of using WB-MRI in assessing disease activity and response to treatment. Persistent pain in children with radiological remission may be attributed to mechanical factors thereby confounding clinical response to treatment [13].

Consensus Treatment Plans developed by The Childhood Arthritis and Rheumatology Research Alliance (CARRA) for the treatment of CNO in patients refractory to NSAIDs and/or with active spinal lesions include methotrexate or sulfasalazine, TNF blockers (with or with- out methotrexate) or bisphosphonates. Use of these Consensus Treatment Plans will provide more information on efficacy in the absence of randomised control trials [3].

Pamidronate was generally well tolerated in our cohort but patients and parents are warned to expect infusion effects and given anti-pyretics as required.

The main limitation of our study is that it is retrospective. Patient related outcomes and functional measures were not recorded at every visit. Our results indicate essentially radiological response to treatment. We also acknowledge that there could have been inter-observer variability in reporting of scans. Few patients received NSAIDs, methotrexate or steroids before pamidronate that could have contributed to the disease outcome.

## Conclusion

From our study we conclude that bisphosphonates seem to be effective and safe in the treatment of CNO especially in the presence of vertebral lesions. WB-MRI is a useful tool in assessing response to treatment. Anti-TNF agents may be used when desired response to bisphosphonates is not achieved. However, further prospective studies are warranted to compare the efficacy of bisphosphonates and anti-TNF agents in the treatment of CNO.

## Data Availability

The datasets used and/or analysed during the current study are available from the corresponding author on reasonable request.

## Abbreviations

CNO: Chronic non-bacterial osteitis
NSAID: Non steroidal anti-inflammatory drugs
STIR: Short tau inversion recovery sequences
TNF: Tumour necrosis factor
UK: United Kingdom
WB MRI: Whole body magnetic resonance imaging
